# Mechanical Properties under Compression and Microscopy Analysis of Basalt Fiber Reinforced Recycled Aggregate Concrete

**DOI:** 10.3390/ma16062520

**Published:** 2023-03-22

**Authors:** Xianggang Zhang, Gaoqiang Zhou, Ping Xu, Lei Fu, Dapeng Deng, Xiaomei Kuang, Yuhui Fan

**Affiliations:** 1 School of Urban Construction, Wuchang University of Technology, Wuhan 430223, China; 2 School of Civil Engineering, Henan Polytechnic University, Jiaozuo 454003, China; 3 Kaifeng Development and Reform Commission, Kaifeng 475000, China

**Keywords:** basalt fiber reinforced recycled aggregate concrete, compression, mechanical properties, conversion relationship, microscopic mechanism

## Abstract

In this study, the basalt fiber content (0%, 0.075%, and 0.15%) and replacement ratio of recycled coarse aggregate (0%, 50%, and 100%) were used as parameters, and the compressive strength of 15 cubes and 15 prisms was analyzed. The failure morphology of the specimens was characterized, and the cubic compressive strength, axial compressive strength, elastic modulus, Poisson’s ratio, and other mechanical property indices of the specimens were measured. Upon increasing the replacement ratio, the degree of damage of the specimens gradually increased, whereas the cubic compressive strength, axial compressive strength, and elastic modulus gradually decreased. As the replacement ratio was increased from 50% to 100%, the cubic compressive strength and elastic modulus were noted to decrease the most by about 9.07% and 9.87%, respectively. On the other hand, the Poisson’s ratio first decreased, followed by an increase. Upon increasing the fiber content, the degree of damage of the specimens was gradually reduced, whereas the cubic compressive strength, axial compressive strength, and elastic modulus gradually increased. As the fiber content increased from 0.075% to 0.15%, the axial compressive strength and elastic modulus increased the most by about 6.65% and 10.19%, respectively. On the other hand, the Poisson’s ratio gradually decreased. Based on the test data, the functional relationships between the strength indices and different variables, as well as the conversion value of each strength index and different variables were established; after comparison and verification, the formula calculation results were found to be in good agreement with the test results. The microstructural changes in the basalt fiber reinforced recycled aggregate concrete were characterized by scanning electron microscopy (SEM), and the changes in the mechanical properties of the basalt fiber reinforced recycled aggregate concrete as well as the mechanism of fiber modification and reinforcement were explained from a micro perspective.

## 1. Introduction

With the acceleration of urbanization, an increasing number of buildings have been demolished, thus resulting in a large amount of construction waste. Simultaneously, the construction of new buildings requires the use of a large amount of natural resources and construction materials. The large resource consumption inevitably causes a significant damage to the environment. Thus, it is of vital concern to effectively deal with waste concrete [1,2,3,4]. Recycled aggregate concrete (RAC) is a new material made up of waste concrete after mechanical or artificial crushing, screening, grading, and other processes, in accordance with the designed proportion attained by partially or completely replacing the natural coarse aggregate (NCA) [5,6,7,8]. Because of the original damage or micro-cracks in the aggregate crushing process [9,10], the bearing capacity of RAC is lower compared with the natural aggregate concrete [11]. The disadvantages of high porosity [12,13], strong water absorption [14], and low aggregate strength [15,16] further affect the development of the RAC technology.

Basalt fiber (BF) represents a green concrete reinforcing material. Compared with the glass fiber [17,18], steel fiber [19,20,21], recycled fiber [22,23], and other materials, BF has relatively stable chemical properties, thermal stability, mechanical properties [24,25], and cost effectiveness. The addition of a certain amount of BF imparts BFRRAC superior mechanical properties [26,27]. Zhang et al. [28] studied the influence of BF content on BFRRAC under different RAC strength grades. It was observed that the optimal fiber content for different strength grades of BFRRAC was different, and the BF content increased with the concrete strength grade. The optimal fiber volume content of C25, C35, and C45 was 0.1%, 0.1–0.15% and 0.15%, respectively. Furthermore, the bonding force between the concrete and fiber increased with the concrete strength grade. Ahmadi et al. [29] studied the use of recycled steel fibers by 0.5% to 1%, and the results showed that adding fibers by 0.5% increased the compressive strength to 10% at the highest value. However, adding fibers by 1% reduced the compressive strength by 12% at the maximum amount. Sun et al. [30] studied the mechanical properties of BFRRAC as a function of fiber content. Upon increasing the fiber content, the compressive strength of the concrete material first increased, followed by a decrease, while the bending strength increased gradually. As the fiber content increased from 0 to 3%, the compressive strength increased the most by about 24.4%, and as the fiber content increased from 0 to 5%, the bending strength increased the most by about 28.2%. Katkhuda and Shatarat [31] and Jiang et al. [32] studied the reinforcing effect of BF on the mechanical properties of RAC as a function of BF content. BF was noted to result in a small increase in compressive strength, along with a significant increment in the transverse and splitting tensile strength. Dong et al. [33] studied the effect of BF on the RAC property. The use of BF improved the concrete strength of the mixtures, and an optimal fiber content in each concrete formulation provided a high strength value, but the maximum decreased by 55.6% and 32.3% in the splitting tensile strength and the flexural strength, respectively. Wang et al. [34] demonstrated that at a BF content of 1 kg/m^3^ and 2 kg/m^3^, upon increasing of the recycled coarse aggregate (RCA) replacement ratio, the axial compressive strength first increased followed by a decrease. Furthermore, the maximum axial compressive strength was obtained as the RCA replacement ratio was 50%. Fang et al. [35] reported that an optimal BF content effectively improved the flexural, splitting tensile, and compressive strength of RAC; however, the excess BF amount led to the formation of agglomerates, thus weakening the mechanical properties of RAC. Compared with the BF fiber content of 0%, when the BF volume fraction was 0.05%, 0.1%, and 0.2%, the cube compressive strengths decreased by 9.9%, 1.0% and 10.6%, respectively. The axial compressive strengths decreased by 3.0%, 5.2%, and 1.7%, respectively. Dilbas and Cakir [36] observed that the physical properties of RAC decreased significantly upon increasing the BF content and RAC replacement ratio, and the effect of BF content on the physical properties was less than the replacement ratio of RAC. At the same time, the incorporation of BF significantly improved the mechanical properties of RAC.

Overall, the literature studies show that the mechanical properties of RAC can be improved by fiber modification. However, only a handful of studies have simultaneously focused on the BFRRAC mechanical properties and microstructure, and only failure modes and test results have been described in most studies and calculation formulas for BFRRAC are rarely proposed. This study aims to close the gap in the literature while providing important outcomes about BFRRAC regarding mechanical properties, and to propose a constitutive relationship suitable for BFRRAC. For this purpose, the mechanical properties of RAC have been studied with BF content and RCA replacement ratio as the variables. At the same time, the characteristics of BFRRAC have been analyzed by SEM after compression failure. The functional relationships between the strength indices and different variables, as well as the conversion value of each strength index and different variables have been established, which provides reference for further research and applications of BFRRAC.

## 2. Methods and Materials

### 2.1. Materials

**Figure 1:**
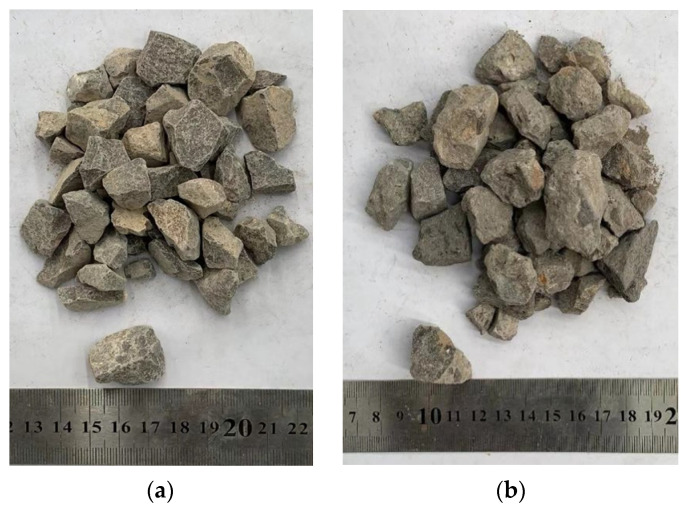
Coarse aggregate: (**a**) Natural coarse aggregate and (**b**) recycled coarse aggregate.

**Figure 2:**
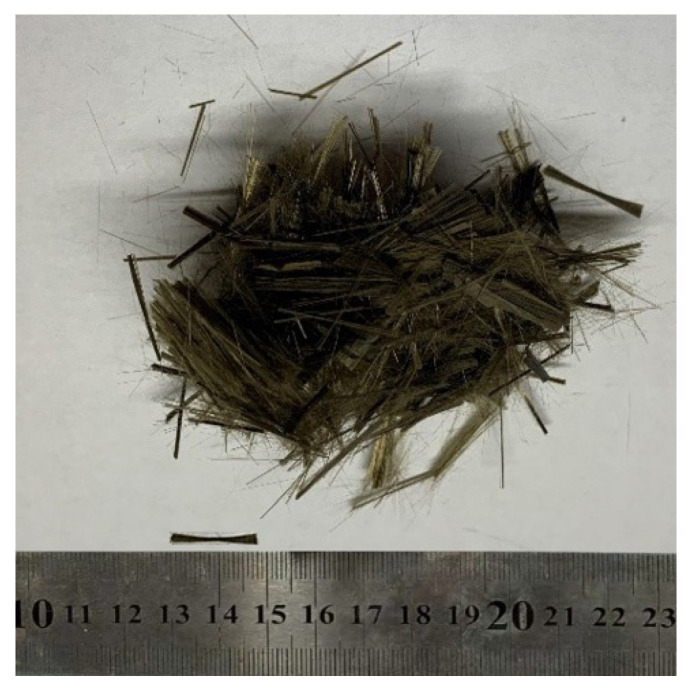
Basalt fiber.

The following materials were used to fabricate the BFRRAC specimens: cement, natural coarse aggregate, recycled coarse aggregate, natural fine aggregate, BF, high-performance polycarboxylic acid superplasticizer, and tap water. Among these, P·O 42.5 Ordinary Portland Cement was selected, and the quality indexes are shown in Table 1. The NCA was natural continuously graded gravel, with a particle size range of 5~31.5 mm. RCA was obtained by artificial crushing, grading, screening, and drying of the wasted reinforced concrete beam from the structural hall of Henan Polytechnic University, with a particle size range 5~31.5 mm, and the design strength grade of original concrete was C25. The coarse aggregates are shown in Figure 1a,b, respectively, and their basic physical properties are shown in Table 2. Natural yellow sand was selected as the fine aggregate. Its bulk and apparent density values were 1750 kg/m^3^ and 2300 kg/m^3^, respectively, and the fineness modulus range was 1.6~2.2. Grade II fly ash produced by a power plant in Gongyi City was used in this study. The basalt fibers are shown in Figure 2, and their quality indices are shown in Table 3.

### 2.2. Design of Mixture Proportions

**Figure 3:**
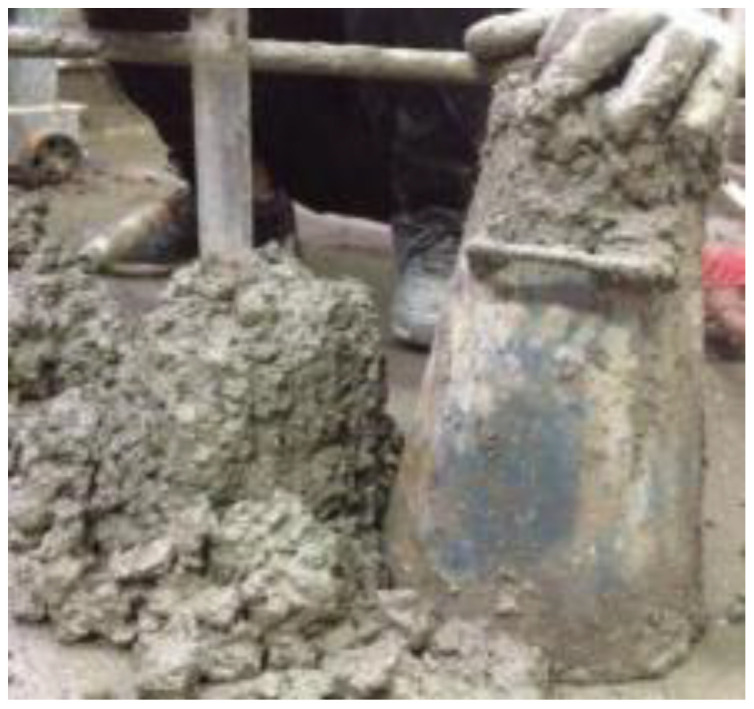
The slump measurement of the BFRRAC mixture.

The RCA replacement ratios were selected to be 0%, 50%, and 100% (weight percentage of RCA to total coarse aggregate), and the BF content was varied as 0 kg/m^3^, 2 kg/m^3^, and 4 kg/m^3^ (the corresponding volume content was 0%, 0.075%, and 0.15%, respectively). The design of mixture proportions is presented in Table 4. The slump of BFRRAC mixture was measured in the range 130~160 mm, as shown in Figure 3. 

### 2.3. Loading and Characterization of Specimens

An electro-hydraulic servo pressure testing machine (WAW-2000) with a maximum test force of 5000 kN was used. The cubic and axial compressive strength tests were carried out per “Standard for test methods of concrete physical and mechanical properties” (China, GB/T50081-2019) [37], with a loading rate of 0.5 MPa/s. The BF content and replacement ratio of RCA were used as the variables. Fifteen cubes with dimensions of 150 mm × 150 mm × 150 mm were fabricated for the cubic compressive strength test, whereas 15 prisms of dimensions 150 mm × 150 mm × 300 mm were fabricated for the axial compression, elastic modulus, and Poisson’s ratio tests.

**Figure 4:**
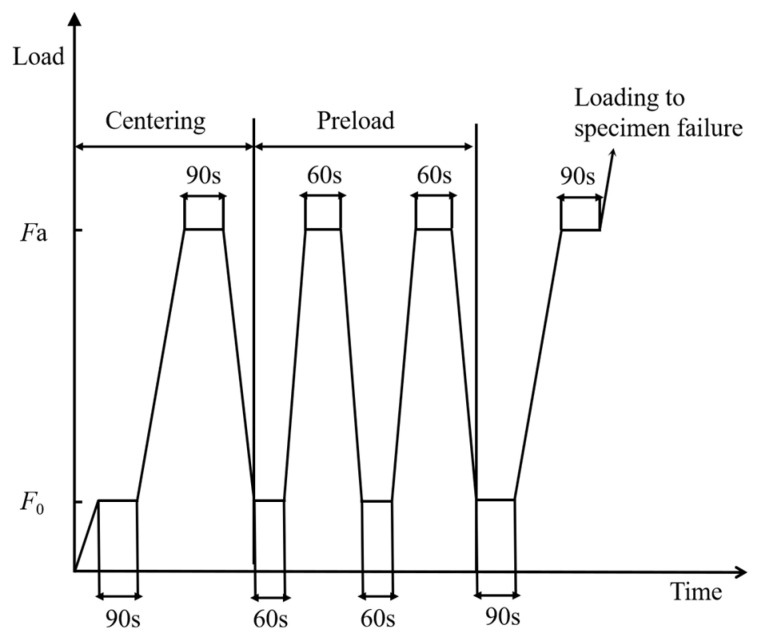
Elastic modulus loading time history.

**Figure 5:**
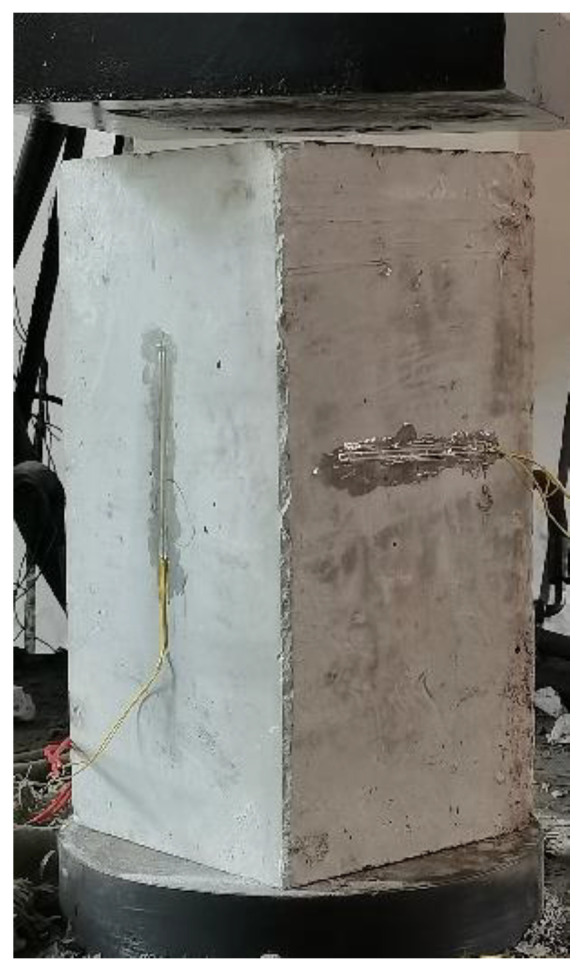
Elastic modulus test.

The test loading method of elastic modulus is shown in Figure 4 and Figure 5. The loading rate of the elastic modulus was 0.5 MPa/s, the reference load value *F*_0_ was 0.5 MPa, and the maximum preload value *F*_a_ was 1/3 of the axial compressive strength of the specimen. After the preload was centered, three repeated preloadings were performed between *F*_0_ and *F*_a_. When loading to *F*_0_ and *F*_a_, the dead load was 60 s, and the deformation value of each measuring point was recorded, and then the elastic modulus was calculated. 

## 3. Results and Discussion

### 3.1. Failure Process and Morphology

#### 3.1.1. Cubic Compression Failure

**Figure 6:**
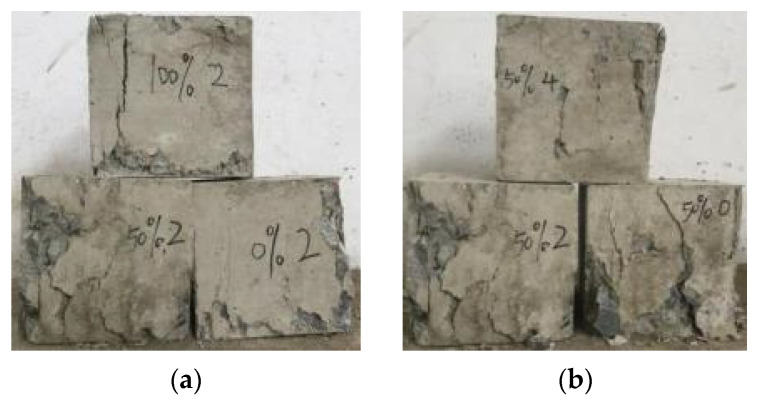
The failure morphology of cubic specimens: (**a**) different replacement ratio (**b**) different BF content.

The cubic compression failure process of BFRRAC specimens was roughly similar to that of the ordinary concrete specimens. At the initial stages of specimen loading, the specimen mainly resisted the increase in the external load by the overall elastic deformation, and the surface had no obvious change. As the load was increased gradually, cracks began to appear in the stress concentration area inside the specimen. Subsequently, the original micro-cracks of the RCA began to develop and increase. As the load continued to increase, the crackling sound of the colloidal cracking appeared, with the expansion of the internal cracks of the specimen. As a result, several vertical cracks appeared in the middle of the side in the direction 45~60°, and the surface of the BFRRAC began to bulge and peel. Upon reaching the ultimate load value, the specimen was eventually destroyed, and the cubic compression of the BFRRAC specimen exhibited a quadrangular pyramid failure morphology, as shown in Figure 6. As can be observed, the failure morphology of the specimens was dependent on the replacement ratio and BF content. At a same BF content, upon increasing the RCA replacement ratio, the number of cracks gradually decreased; however, the width became large. Furthermore, the degree of brittle failure of the specimens became more obvious. Keeping the RCA replacement ratio constant, upon increasing the BF content, a large number of unpenetrated fine cracks were observed after the failure of the specimen. Although the specimen was cracked; however, it did not scatter into fragments. The upper and lower parts of the specimen were noted to be basically intact, whereas the middle phase exhibited the presence of defects. Thus, increasing the BF content was noted to significantly improve the brittleness of BFRRAC.

#### 3.1.2. Axial Compression Failure

**Figure 7:**
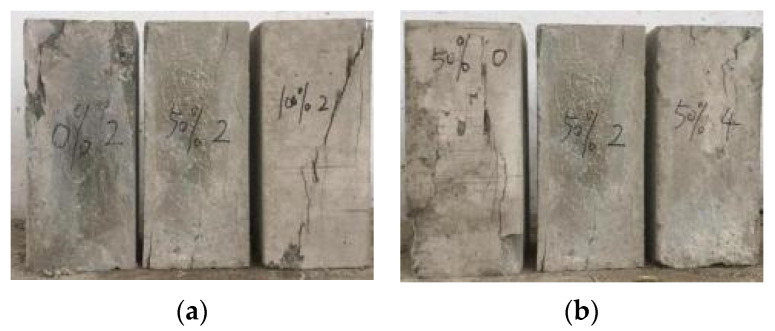
The failure morphology of prism specimens: (**a**) different replacement ratio (**b**) different BF content.

At the initial stages of specimen loading, the surface of the BFRRAC prism did not exhibit any obvious change. As the load increased gradually, the specimen expanded laterally, and the original micro-cracks existing in the RCA began to develop. Furthermore, the micro-cracks between the new/old mortar and RCA began to expand upon increasing the load. BF distributed randomly inside the specimen could alleviate the stress concentration at the crack tip, thus preventing the crack propagation to a certain extent and balancing the internal force. As a result, the generation and development of cracks tended to be relatively stable. As the load continued to increase, the width and number of cracks increased sharply. The shear stress in the 45° direction reached the limit value, and the specimen exhibited the shear slip failure along the oblique section. A fraction of concrete fell off, and the internal aggregate was exposed, thus the bearing capacity was reduced. At this stage, the cracks were unstable and exhibited a sustainable development without increasing the external load. Afterwards, upon increasing the external load, the bonding between the cement mortar at the main crack, aggregate, and BF was lost, and the friction bite force on the slip surface was basically exhausted. Finally, the specimen completely lost its bearing capacity. The morphology of the axial compression of the BFRRAC specimen is shown in Figure 7. As can be observed, the integrity of each specimen was superior after failure. Chen et al. [38] also proposed that BF fiber has a significant toughening effect, the integrity of the specimen is good after failure, and only a small number of particles fall off. Furthermore, mortar loss was observed on the surface of a few specimens, and the surface of the peeled BFRRAC was irregular. 

### 3.2. Test Result

The compression property indices of BFRRAC are shown in Table 5. It can be observed that the cubic and axial compressive strength decreased upon increasing the replacement ratio, whereas these were observed to increase with the BF content.

### 3.3. Analysis of Influencing Factors

#### 3.3.1. Compressive Strength

**Figure 8:**
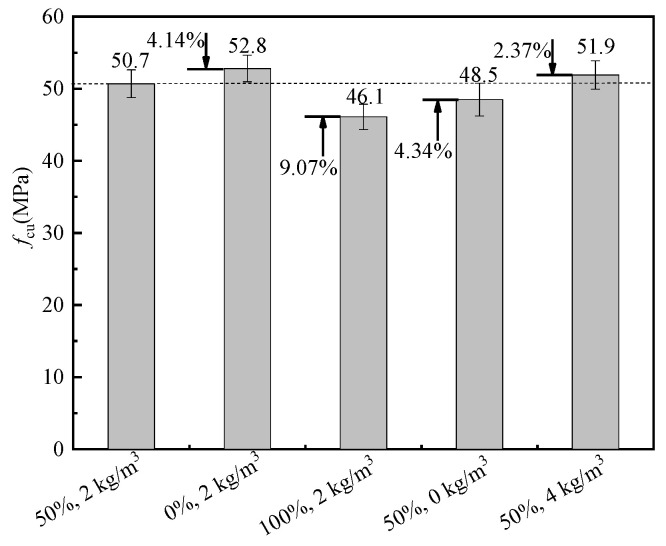
Comparison of cubic compressive strength.

**Figure 9:**
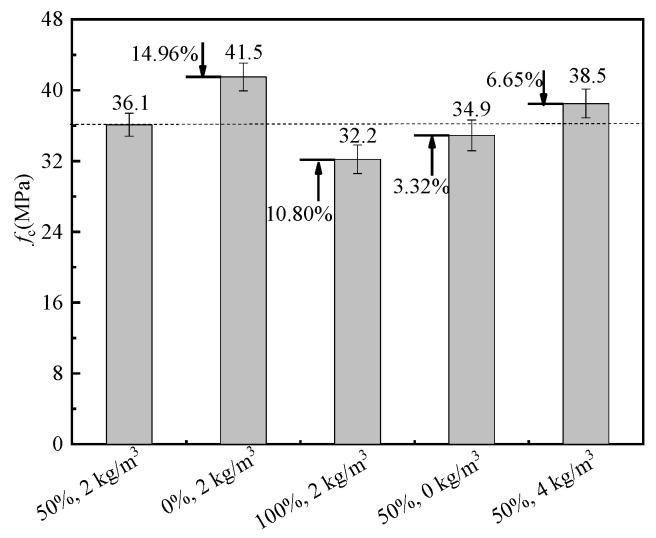
Comparison of axial compressive strength.

The cubic and axial compressive strength of BFRRAC as a function of different variables are shown in Figure 8 and Figure 9, respectively. Compared with the benchmark specimen with 50% RCA replacement ratio and 2 kg/m^3^ BF content, at a BF content of 2 kg/m^3^ with the RCA replacement ratios changed to 0% and 100%, the cubic compressive strength increased by 4.14% and decreased by 9.07%, respectively, whereas the axial compressive strength increased by 14.96% and decreased by 10.80%, respectively. Wang et al. [39] and Zheng et al. [40] also proposed that the addition of an appropriate amount of basalt fiber could improve the compressive strength of RAC. The observed phenomenon was mainly due to a large number of micro-cracks inside RCA after crushing and the old mortar attached to the RCA surface. The original damage accumulated upon increasing the replacement ratio, which was reflected by a decline in strength. At an RCA replacement ratio of 50%, the BF content changed to 0 kg/m^3^ and 4 kg/m^3^, and the cubic compressive strength decreased by 4.34% and increased by 2.37%, respectively, whereas the axial compressive strength decreased by 3.32% and increased by 6.65%, respectively. The observed phenomenon was attributed to the loading process, which enhanced the axial deformation of the specimen and the bridging effect of BF effectively prevented the expansion of the cracks, thus the strength of the specimen was improved. In summary, the cubic and axial compressive strength of BFRRAC decreased upon increasing the RCA replacement ratio, whereas these exhibited an increase with BF content. Moreover, changing the RCA replacement ratio had a greater impact on the compressive strength of the specimens than the BF content.

#### 3.3.2. The Functional Relationships between *f*_cu_, *f*_c_ and Different Variables

**Figure 10:**
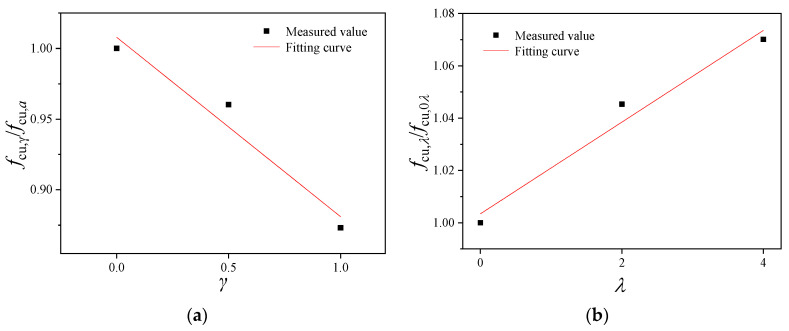
The fitting curve of the cubic compressive strength of specimens under different variable parameters: (**a**) fitting curve of *f*_cu,γ_/*f*_cu,*a*_ and *γ* (**b**) fitting curve of *f*_cu,*λ*_/*f*_cu,*b*_ and *λ*.

Based on the measured values in Table 4, the compressive strength of the cubic specimens was subjected to dimension normalization and fitting, as shown in Figure 10. It can be seen that the dimensionless cubic compressive strength of BFRRAC showed a linear development trend with the RAC replacement ratio and BF content. Under the condition of a single parameter change (either replacement ratio or BF content), by using the least squares method, Equations (1) and (2), respectively, were obtained:

(1) $$f_{\mathrm{cu},\gamma }/f_{\mathrm{cu},a}=-0.127\gamma +1.008\;\;\;\;\;\;\;\;\;\;\;\;\;\;\;\;\;\;\;\;\;\;\;\;\;\;\;\;\;R^{2}=0.96$$

(2) $$f_{\mathrm{cu},\lambda }/f_{\mathrm{cu},b}=0.018\lambda +1.003\;\;\;\;\;\;\;\;\;\;\;\;\;\;\;\;\;\;\;\;\;\;\;\;\;\;\;\;\;\;R^{2}=0.97$$

where the replacement ratio of RCA is represented by *γ*, the BF content is represented by *λ*, *f*_cu,*γ*_ represents the cubic compressive strength values of BFRRAC with a BF content of 2 kg/m^3^ under different RCA replacement ratios, *f*_cu,*a*_ represents the cubic compressive strength value of BFRRAC at a BF content of 2 kg/m^3^ and a RCA replacement ratio of 0%, *f*_cu,*λ*_ represents the cubic compressive strength values of BFRRAC with a RCA replacement ratio of 50% under different BF contents, and *f*_cu,*b*_ represents the cubic compressive strength of BFRRAC at a BF content of 0 kg/m^3^ and a RCA replacement ratio of 50%.

**Figure 11:**
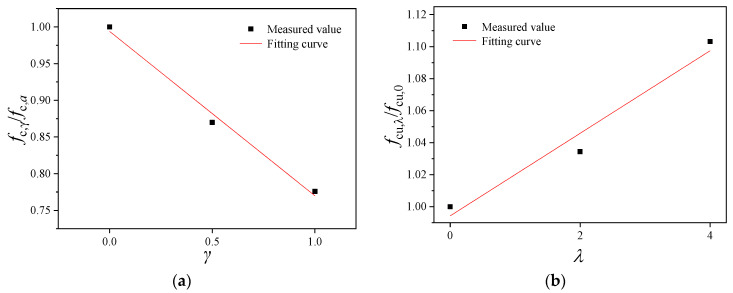
The fitting curve of the axial compressive strength of specimens under different variable parameters: (**a**) fitting curve of *f*_c,*γ*_/*f*_c,*a*_ and *γ* and (**b**) fitting curve of *f*_c,*λ*_/*f*_c,*b*_ and *λ*.

Based on the measured values in Table 4, the compressive strength of the prism specimens was subjected to dimension normalization and fitting, as shown in Figure 11. It can be seen that the dimensionless axial compressive strength of BFRRAC exhibited a linear development trend with the RAC replacement ratio and BF content. Under the condition of a single parameter change (either replacement ratio or BF content), by using the least squares method, Equations (3) and (4), respectively, were obtained:

(3) $$f_{c,\gamma }/f_{c,a}=-0.224\gamma +0.994\;\;\;\;\;\;\;\;\;\;\;\;\;\;\;\;\;\;\;\;\;\;\;\;\;\;\;\;\;\;R^{2}=0.99$$

(4) $$f_{c,\lambda }/f_{c,b}=0.026\lambda +0.994\;\;\;\;\;\;\;\;\;\;\;\;\;\;\;\;\;\;\;\;\;\;\;\;\;\;\;\;\;\;R^{2}=0.96$$

where *f*_c,*γ*_ represents the axial compressive strength values of BFRRAC at a BF content of 2 kg/m^3^ under different RCA replacement ratios, *f*_c,*a*_ represents the axial compressive strength of BFRRAC at a BF content of 2 kg/m^3^ and a RCA replacement ratio of 0%, *f*_c,*λ*_ represents the axial compressive strengths of BFRRAC at a RCA replacement ratio of 50% under different BF contents, and *f*_c,*b*_ represents the axial compressive strength of BFRRAC at a BF content of 0 kg/m^3^ and a RCA replacement ratio of 50%.

#### 3.3.3. *f*_c_/*f*_cu_

**Figure 12:**
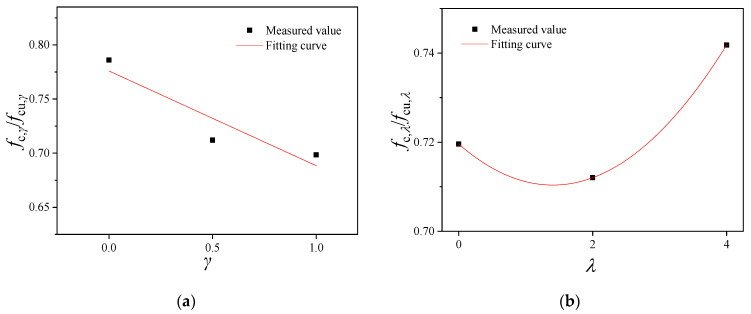
The fitting curve of the relation between the cubic compressive strength and the axial compressive strength of the specimen: (**a**) fitting curve of *f*_c,*γ*_/*f*_cu,*γ*_ and *γ* and (**b**) fitting curve of *f*_c,*λ*_/*f*_cu,*λ*_ and *λ*.

Because of the influence of the BF content and replacement ratio, the conversion relationship between the cubic and axial compressive strength of the ordinary concrete is no longer applicable to RAC. Therefore, the relation between the cubic and the axial compressive strength of the specimen was subjected to fitting, as shown in Figure 12. The functional relationships between the conversion value of each strength index and different variables were established, as shown in the Equations (5) and (6), respectively.

(5) $$f_{c,\gamma }/f_{\mathrm{cu},\gamma }=-0.088\gamma +0.776\;\;\;\;\;\;\;\;\;\;\;\;\;\;\;\;\;\;\;\;\;\;\;\;\;\;\;\;\;\;R^{2}=0.86$$

(6) $$f_{c,\lambda }/f_{\mathrm{cu},\lambda }=0.005\lambda^{2}-0.013\lambda +0.72\;\;\;\;\;\;\;\;\;\;\;\;\;\;\;\;\;\;\;\;\;\;\;\;\;\;\;\;\;\;R^{2}=1.00$$

where *f*_c,*γ*_ and *f*_cu,*γ*_ represent the axial and cubic compressive strength values of BFRRAC at a BF content of 2 kg/m^3^ BF under different RCA replacement ratios, *f*_c,*λ*_ and *f*_cu,*λ*_ represent the axial and the cubic compressive strength values of BFRRAC at an RCA replacement ratio of 50% under different BF contents.

**Figure 13:**
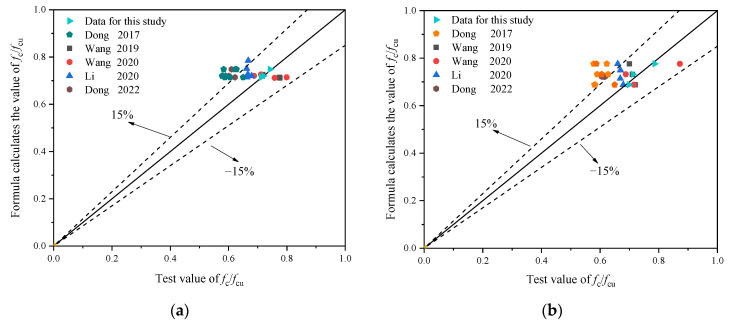
The comparison between the *f*_c_/*f*_cu_ test and the formula calculation results: (**a**) different BF content of RP [33,34,39,41,42] and (**b**) different replacement ratio of RP [33,34,39,41,42].

To further verify the applicability of the calculation formula, the compressive strength of specimens under different BF contents [33,34,39,41,42] and different replacement ratios [33,34,39,41,42] were substituted into the formula calculation, the comparison between the *f*_c_/*f*_cu_ test result and the calculation result based on Equations (5) and (6) is shown in Figure 13a,b, respectively. It can be observed that the formula calculation results were in good agreement with the test results.

#### 3.3.4. Elastic Modulus and Poisson’s Ratio

**Figure 14:**
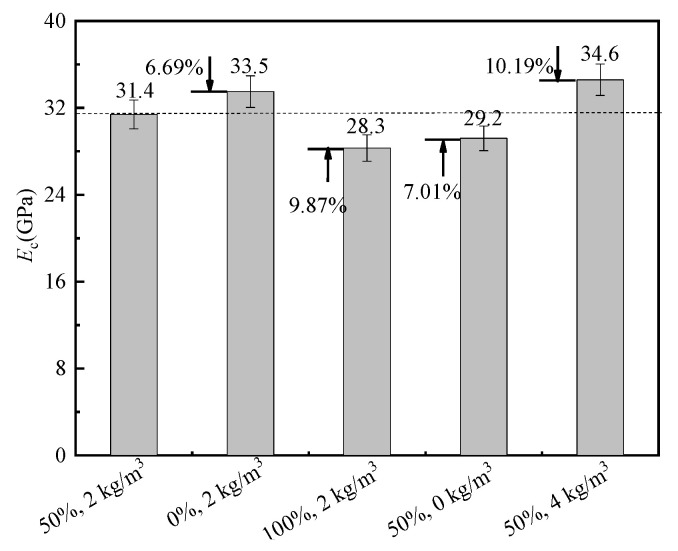
Comparison of the elastic modulus.

**Figure 15:**
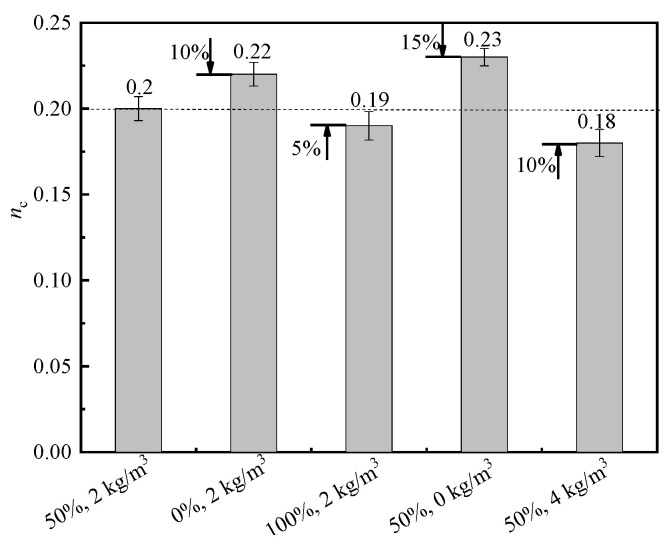
Comparison of the Poisson’s ratio.

The elastic modulus and Poisson’s ratio of BFRRAC as a function of different variables are shown in Figure 14 and Figure 15, respectively. Compared with the benchmark specimen with a 50% RCA replacement ratio and a BF content of 2 kg/m^3^, at a BF content of 2 kg/m^3^ with the RCA replacement ratios changed to 0% and 100%, the elastic modulus increased by 6.69% and decreased by 9.87%, respectively, whereas the Poisson’s ratio increased by 10% and 15%, respectively. At an RCA replacement ratio of 50% with the BF content changed to 0 kg/m^3^ and 4 kg/m^3^, the elastic modulus decreased by 7.01% and increased by 10.19%, respectively, whereas the Poisson’s ratio increased by 15% and decreased by 10%, respectively. In summary, upon increasing the RCA replacement ratio, the elastic modulus of BFRRAC decreased, whereas the Poisson’s ratio first decreased, followed by an increase. This was mainly due to a large number of micro-cracks inside RCA, thus it was easy to expand the crack during the test, and the density of concrete was reduced due to the adhesion of loose and porous old mortar outside RCA. Upon increasing the BF content, the elastic modulus of the prepared BFRRAC gradually increased, while the Poisson’s ratio was observed to decrease. The observed phenomenon was due to the reason that RCA adhered to the old mortar, which enhanced the friction force and mechanical bite force between the BF and mortar matrix. The distribution of BF in BFRRAC was disordered in three dimensions, which played a role in pulling the aggregate and preventing the crack propagation, thereby reducing the lateral deformation of the specimens and reducing the Poisson’s ratio. BF was noted to have an optimal compatibility with RAC. Upon incorporating a certain amount of BF, the compactness of the specimen and the continuity of the matrix increased, thus the impact of the original damage caused by crushing decreased, which enhanced the elastic modulus of the specimen.

The elastic modulus is a vital index reflecting the deformation ability of the materials. The elastic modulus of BFRRAC has been studied in many literature studies [36,41]. However, the mathematical expressions of the elastic modulus have been rarely proposed. Based on the “Code for design of concrete structures (China, GB 50010-2010)” [43], the calculation of the elastic modulus of the ordinary concrete was performed as per Equation (7).

(7) $$E_{c}{}^{{}_{c}}=\frac{10^{5}}{2.2+\frac{34.7}{f_{\mathrm{cu}}}}$$

**Figure 16:**
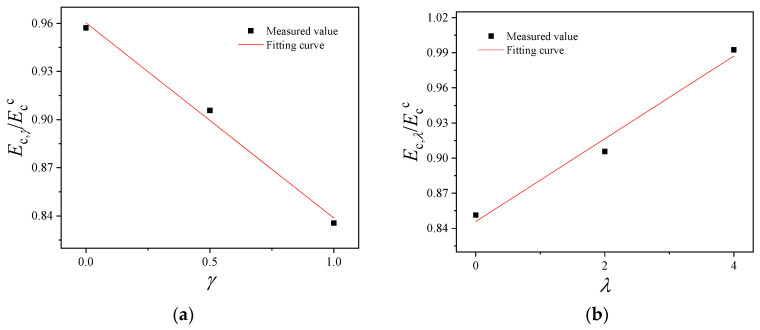
The elastic modulus and the fitting curve under different variable parameters: (**a**) fitting curve of *E*_c,_*_γ_*/*E*_c_^c^ and *γ* and (**b**) fitting curve of *E*_c,*λ*_/*E*_c_^c^ and *λ*.

The measured data were substituted in Equation (7) to obtain the calculated elastic modulus (*E*_c_^c^) of BFRRAC. The fitting curves of (*E*_c_) and (*E*_c_^c^) as a function of different parameters are shown in Figure 16. As can be observed, the calculation as per the “Code for design of concrete structures (China, GB/T50010-2010)” generally provided a greater *E*_c_^c^ value than *E*_c_. Obviously, the current code was not applicable to the calculation of the elastic modulus of BFRRAC, and *E*_c_/*E*_c_^c^ showed a linear increasing trend with the RAC replacement ratio and BF content. Under the condition of a single parameter change (either replacement ratio or BF content), by using the least squares method, Equations (8) and (9), respectively, were obtained:

(8) $$E_{c,\gamma }=\frac{10^{5}}{2.2+\frac{34.7}{f_{\mathrm{cu}}}}(-0.122\gamma +0.960\;)\;\;\;\;\;\;\;\;\;\;\;\;\;\;\;\;\;\;\;\;\;\;\;\;\;\;\;\;\;\;\;R^{2}=0.99$$

(9) $$E_{c,\lambda }=\frac{10^{5}}{2.2+\frac{34.7}{f_{\mathrm{cu}}}}(0.035\lambda +0.846\;\;)\;\;\;\;\;\;\;\;\;\;\;\;\;\;\;\;\;\;\;\;\;\;\;\;\;\;\;\;\;\;R^{2}=0.98$$

where *E*_c,*γ*_ represents the calculated elastic modulus of BFRRAC with a BF content of 2 kg/m^3^ under different RCA replacement ratios, and *E*_c,*λ*_ represents the calculated elastic modulus of BFRRAC with an RCA replacement ratio 50% under different BF contents.

**Figure 17:**
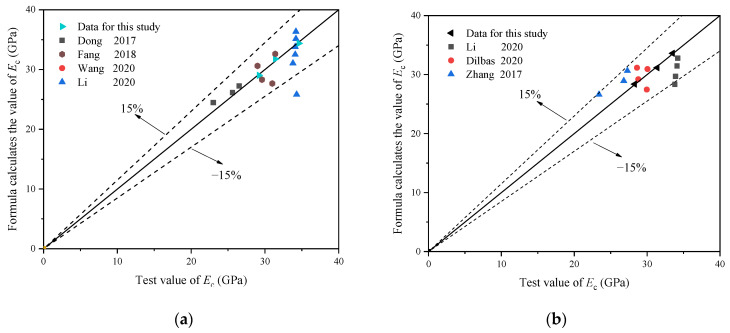
The comparison between the test and the formula calculation results of *E*_c_: (**a**) different BF content of RP [33,35,39,41] and (**b**) different replacement ratio of RP [28,41,44].

To further verify the applicability of the calculation formula, the specimen data under different BF contents [33,35,39,41] and different replacement ratios [28,41,44] were substituted into the formula calculation; the comparison between the test result and the formula calculation result of *E*_c_ based on Equations (8) and (9) is shown in Figure 17a and Figure 17b, respectively. It can be observed that the formula calculation results were in good agreement with the test results.

### 3.4. Analysis of Variance

The significance and primary and secondary order of the influence of each factor on the performance index were reflected by the analysis of variance (ANOVA). In addition, the influence relationship and law of each factor on the performance index were analyzed. In this study, the analysis was performed at a 5% level of significance to identify the statistically significant experimental parameters. For this, the data presented in Table 5 were examined by using the general linear model analysis of variance technique by means of software called “SPSS”. The cubic and axial compressive strength were assigned as dependent variables while the BF content and the replacement ratio of the recycled coarse aggregate (RCA) were selected as independent factors. The statistical analysis results are shown in Table 6. The *p*-values indicate the significance of the test parameter on the cubic and axial compressive strength. When the *p*-value was less than 5%, the parameter had a significant effect on the cubic and axial compressive strength. The contribution ratio represented the degree of response of each influencing factor to the dependent variable. According to the statistical analysis results, the contribution of the BF fiber content replacement ratio of the recycled coarse aggregate could be underestimated when compared with the contribution of the replacement ratio of the recycled coarse aggregate.

### 3.5. Microscopy Analysis of BFRRAC

**Figure 18:**
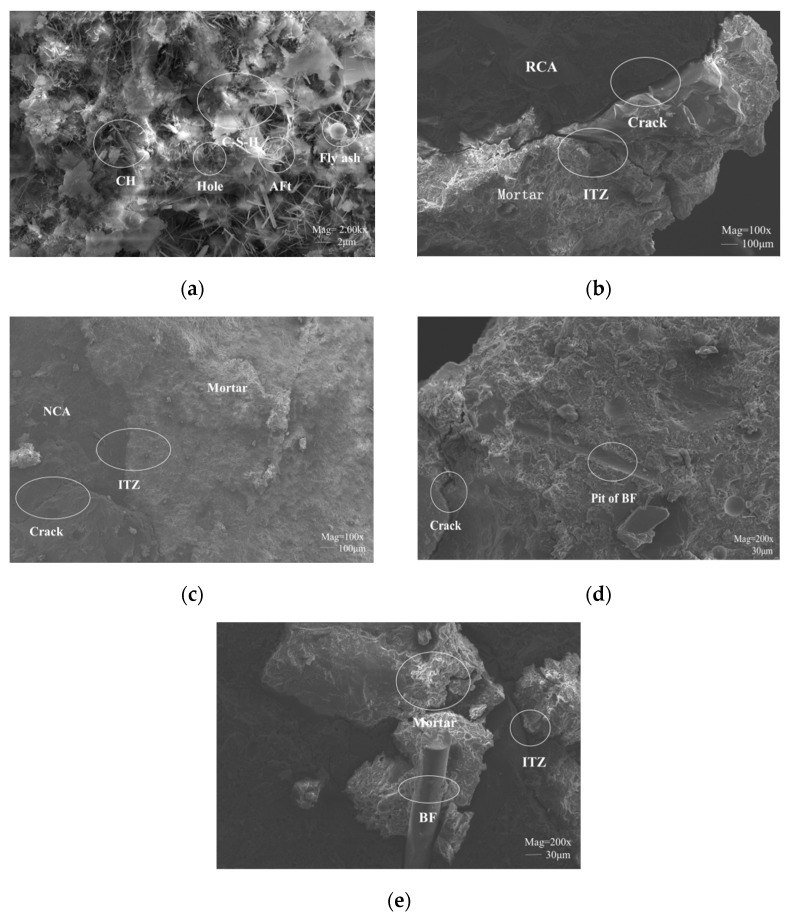
Micromorphology of BFRRAC: (**a**) mortar matrix, (**b**) recycled aggregate concrete, (**c**) natural aggregate concrete, (**d**) pit of BF, and (**e**) failure morphology of BF.

The SEM analysis of BFRRAC is presented in Figure 18 (image magnification is represented by Mag, and the scale length is represented by the white lines).

As shown in Figure 18a, the micromorphology of the BFRRAC mortar matrix was mainly composed of the colloidal substances, such as the hydrated calcium silicate gel (C-S-H) of the flocculent, calcium hydroxide gel (CH) of the layered, and ettringite (AFt) of the needle columnar. The hydration products were noted to be tightly bound, which ensured that the specimen had superior mechanical properties. The hydration products appeared on the surface of fly ash, which gradually participated in the secondary hydration reaction. The fly ash also reacted with other basic hydroxides such as CH to produce C-S-H and other compounds with water-hardening cementitious properties, thus making the mortar matrix structure more compact with improved macroscopic mechanical properties.

The micromorphologies of the interfacial transition zone (ITZ) between RCA or NCA and mortar matrix are shown in Figure 18b,c, respectively. Overall, the mortar matrix was noted to be dense. The bonding between NCA and mortar matrix was observed to be firm; however, a small number of micro-cracks were present between the RCA and mortar matrix. An obvious presence of ITZ was noted between the aggregate and mortar matrix, and the ITZ between the RCA and mortar matrix was observed to be weaker than that between the NCA and mortar matrix. The microcracks in RAC may have appeared due to the external forces as the recycled aggregate was broken. On the other hand, as the test was carried out, the cracks further expanded under the pressure. Similarly, the conclusion of Dong et al. [33] also proves that the ITZ is an important factor affecting the strength of RAC. Therefore, the mechanical properties of RAC were noted to be inferior compared with NAC.

As shown in Figure 18d,e, there was an obvious ITZ between the BF of the bridge cracks and mortar matrix after the concrete cracks. The mortar matrix was noted to be relatively dense, which indicates that BF had an optimal bridging effect in RAC. This is consistent with the results of Zheng et al. [40], when the fiber content was less than 0.2%, with the increase in fiber content, the mortar matrix became denser and denser. The toughening and crack resistance of BF could effectively prevent the development of cracks and improve the brittleness of the mortar matrix, thus optimizing the macroscopic mechanical properties of the specimen and improving the overall toughness. The morphology of BF being pulled out and failure indicate that BF not only prevented the development of the micro-cracks and cracks, but that is also bore a fraction of the stress, thus contributing towards the improvement of the macroscopic mechanical properties.

## 4. Conclusions

This study investigated the basic mechanical properties of BFRRAC at the macroscopic and microscopic levels, along with discussing the influence of the RCA replacement ratio and BF content on the compressive failure morphology, strength, and deformation of BFRRAC. The main conclusions were as follows:

- The morphology of the cubic specimens in each group under compression were noted to be similar, representing quadrangular pyramid failure. The BFRRAC prisms in each group represented typical oblique section shear failure, and the incorporation of BF enhanced the integrity of each specimen after failure.
- The cubic and axial compressive strength of BFRRAC decreased upon increasing the RCA replacement ratio, whereas these increased with the BF content. As the replacement ratio was increased from 50% to 100%, the cubic compressive strength was noted to decrease the most, by about 9.07%. As the fiber content was increased from 2 kg/m^3^ to 4 kg/m^3^, the axial compressive strength increased the most, by about 6.65%. In addition, the influence of the RAC replacement ratio on the compressive strength of the specimens was noted to be greater than that of the BF content.
- Upon increasing the RCA replacement ratio, the elastic modulus of BFRRAC decreased, whereas the Poisson’s ratio first decreased followed by an increase. As the replacement ratio was increased from 50% to 100%, the elastic modulus was noted to decrease the most, by about 9.87%. Upon increasing the BF content, the elastic modulus of BFRRAC increased, and the Poisson’s ratio decreased. As the fiber content was increased from 2 kg/m^3^ to 4 kg/m^3^, the elastic modulus increased the most by about 10.19%.
- Based on the experiment data, the functional relationships between the strength indices and different variables as well as the conversion value of each strength index and different variables were established. In addition, by comparing the test data in the literature, the formula calculation results were in good agreement with the test results and could provide guidance for future applications in engineering.
- The micromorphology of the mortar matrix and ITZ, as well as the modification mechanism of BF directly affected the macroscopic mechanical properties of RAC. The mechanical properties of RAC could be optimized by the bridging and crack resistance mechanism of BF.
- Overall, this study carried out the mechanical properties test and microscopy analysis of BFRRAC, and established the relationship between different parameters and BFRRAC. In the future, we can continue to carry out in-depth research on this idea: Visualization of fiber distribution and pore structure distribution of RAC is studied by numerical simulation software and the three-dimensional microscopic analysis of dynamic and static mechanical properties of BFRRAC.

## Figures and Tables

**Table 1:** The quality indexes of ordinary Portland cement Grade 42.5.

Fineness (%)	Ignition Loss (%)	SO_3_ (%)	MgO (%)	*f*_cu_ (MPa)	*f*_cf_ (MPa)	Initial Setting Time (min)	Final Setting Time (min)
1.30	2.41	2.45	2.11	51	8.5	180	265

**Table 2:** Basic physical properties of coarse aggregate.

Aggregate Type	Particle Size (mm)	Bulk Density (kg/m^3^)	Apparent Density (kg/m^3^)	Water Content (%)	Water Absorption (%)	Water Content (%)
Natural	5~31.5	1681	2498	0.0	0.1	0.0
Recycled	5~31.5	1274	2196	2.0	5.6	0.0

**Table 3:** Quality indices of BF.

Diameter (μm)	Length (mm)	Density (kg/m^3^)	Tensile Strength (MPa)	Elastic Modulus (GPa)	Elongation at Break (%)
15	18	2650	4150~4850	93~115	3.0~3.2

**Table 4:** Mixture proportion of RAC (kg/m^3^).

*γ* (%)	*W*/*B*	Sand Ratio (%)	Net Water	Aditional Water	Cement	Fly Ash	Recycled Coarse Aggregate	Natural Coarse Aggregate	Sand	Water Reducer
0	0.40	31	205	0.0	427.1	85.4	0.0	1115.2	501	2.56
50	0.40	31	205	31.2	427.1	85.4	557.6	557.6	501	2.56
100	0.40	31	205	62.5	427.1	85.4	1115.2	0.0	501	2.56

Note: Recycled course aggregate replacement ratio represented by *γ*. The dosage of fly ash is 20% of the cement content. The water reducer is 0.5% of the cementitious materials (cement and fly ash).

**Table 5:** Property indices of BFRRAC.

*γ* (%)	*λ* (kg/m^3^)	*f*_cu_ (MPa)	*f*_c_ (MPa)	*E*_c_ (GPa)	*ν*_c_
0	2	52.8	41.5	33.5	0.22
50	0	48.5	34.9	29.2	0.23
	2	50.7	36.1	31.4	0.20
	4	51.9	38.5	34.6	0.18
100	2	46.1	32.2	28.3	0.23

Note: The volume content corresponding to 0, 2, and 4 kg/m^3^ is 0%, 0.075%, and 0.15%, respectively. The BF content is expressed by *λ*. The cubic compressive strength is expressed by *f*_cu_. The axial compressive strength is expressed by *f*_c_. The elastic modulus is expressed by *E*_c_, and its value is the ratio value of stress to strain at 0.5 *f*_c_ on the stress−strain curve of BFRRAC. The Poisson’s ratio is expressed by *ν*_c._

**Table 6:** Results of ANOVA for cubic and axial compressive strength.

Dependent Variable	Independent Variable	Degrees of Freedom	Sum of Squares	Variance	*F*	*p*	Contribution (%)
*f*_cu_	BF content	2	17.009	8.505	3.788	0.060	11.44
	*γ*	2	69.946	34.973	15.579	0.001	59.83
	Error	10	22.449	2.245			28.73
	Total	14	109.404				100
*f*_c_	BF content	2	19.265	9.633	6.172	0.018	9.76
	*γ*	2	130.485	65.242	41.806	0.000	77.03
	Error	10	15.606	1.561			13.21
	Total	14	165.356				100

## Data Availability

The data used to support the findings of this study are included within the article.
